# Genetic Diversity of IFγ, IL1β, TLR2, and TLR8 Loci in Pulmonary Tuberculosis in Kazakhstan

**DOI:** 10.5195/cajgh.2014.181

**Published:** 2014-12-12

**Authors:** Dauren Yerezhepov, Axat Zhabagin, Ayken Askapuli, Saule Rakhimova, Zhannur Nurkina, Aliya Abilmazhinova, Alma Akhmetova, Askhat Molkenov, Ulykbek Kairov, Ainur Akilzhanova

**Affiliations:** Center for Life Sciences, Nazarbayev University, Astana, Kazakhstan

**Keywords:** tuberculosis, inflammation polymorphism, Kazakhstan

## Abstract

**Introduction:**

Tuberculosis (TB) is caused by bacterium *Mycobacterium tuberculosis* (MTB), and according to the WHO, up to 30% of world population is infected with latent TB. Pathogenesis of TB is multifactorial, and its development depends on environmental, social, microbial, and genetic factors of both the bacterium and the host. The number of TB cases in Kazakhstan has decreased in the past decade, but multidrug-resistant (MDR) TB cases are dramatically increasing. Polymorphisms in genes responsible for immune response have been associated with TB susceptibility. The objective of this study was to investigate the risk of developing pulmonary TB (PTB) associated with polymorphisms in several inflammatory pathway genes among Kazakhstani population.

**Methods:**

703 participants from 3 regions of Kazakhstan were recruited for a case-control study. 251 participants had pulmonary TB (PTB), and 452 were healthy controls (HC). Males and females represented 42.39% and 57.61%, respectively. Of all participants, 67.4% were Kazakhs, 22.8% Russians, 3.4% Ukrainians, and 6.4% were of other origins. Clinical and epidemiological data were collected from medical records, interviews, and questionnaires. DNA samples were genotyped using TaqMan assay on 4 polymorphisms: IFNγ (rs2430561) and IL1β (rs16944), TLR2 (rs5743708) and TLR8 (rs3764880). Statistical data was analyzed using SPSS 19.

**Results:**

Genotyping by IFγ, IL1β, TLR2 showed no significant association with PTB susceptibility (*p* > 0.05). TLR8 genotype A/G was significantly higher in females (F/M – 41.5%/1.3%) and G/G in males (M/F – 49%/20.7%) (χ2=161.43, *p* < 0.001). A significantly increased risk of PTB development was observed for TLR A/G with an adjusted OR of 1.48 (95%, CI: 0.96 – 2.28), and a protective feature was revealed for TLR8 G/G genotype (OR: 0.81, 95%, CI: 0.56 – 1.16, *p* = 0.024). Additional grouping by gender revealed that TLR8 G/G contributes as protective genotype (OR: 1.83, 95%, CI: 1.18 – 2.83, *p* = 0.036) in males of the control group.

**Conclusion:**

Results indicate that heterozygous genotype A/G of TLR8 increases the risk of PTB development, while G/G genotype may serve as protection mechanism. A/A genotype is strongly associated with susceptibility to PTB. To clarify the role of other polymorphisms in susceptibility to PTB in Kazakhstani population, further investigations are needed.

